# Gene expression profiles of novel caprine placental prolactin-related proteins similar to bovine placental prolactin-related proteins

**DOI:** 10.1186/1471-213X-7-16

**Published:** 2007-03-15

**Authors:** Koichi Ushizawa, Toru Takahashi, Misa Hosoe, Keiichiro Kizaki, Yasuyuki Abe, Hiroshi Sasada, Eimei Sato, Kazuyoshi Hashizume

**Affiliations:** 1 Reproductive Biology Research Unit, Division of Animal Sciences, National Institute of Agrobiological Sciences, 2 Ikenodai, Tsukuba, Ibaraki 305-8602, Japan; 2 Department of Veterinary Medicine, Faculty of Agriculture, Iwate University, 3-18-8 Ueda, Morioka, Iwate 020-8550, Japan; 3 Laboratory of Animal Reproduction, Graduate School of Agricultural Sciences, Tohoku University, 1-1 Tsutsumidori-amamiyamachi, Aoba-ku, Sendai, Miyagi 981-8555, Japan

## Abstract

**Background:**

This study reports the identification of a full-length cDNA sequence for two novel caprine prolactin-related proteins (cPRP1 and cPRP6), and their localization and quantitative expression in the placenta. Caprine PRPs are compared with known bovine PRPs. We examined their evolution and role in the ruminant placenta.

**Results:**

Full-length cPRP1 and cPRP6 cDNA were cloned with a 717- and 720- nucleotide open-reading frame corresponding to proteins of 238 and 239 amino acids. The cPRP1 predicted amino acid sequence shares a 72% homology with bovine PRP1 (bPRP1). The cPRP6 predicted amino acid sequence shares a 74% homology with bovine PRP6 (bPRP6). The two cPRPs as well as bPRPs were detected only in the placentome by RT-PCR. Analysis by in situ hybridization revealed the presence of both cPRPs mRNA in the trophoblast binucleate cells. These mRNA were quantified by real-time RT-PCR analysis of the placentome at 30, 50, 90 and 140 days of pregnancy. Both new cPRP genes were able to translate a mature protein in a mammalian cell-expression system. Western blotting established the molecular sizes of 33 kDa for cPRP1 with FLAG-tag and 45 kDa for cPRP6 with FLAG-tag. The sequence properties and localized expression of cPRP1 and cPRP6 were similar to those of bovine. However, their expression profiles differed from those in bovine placenta. Although this study demonstrated possible roles of PRPs in caprine placenta, PRPs may regulate binucleate-cell functions like those in bovine, but their crucial roles are still unclear.

**Conclusion:**

We have identified the novel PRPs in caprine placenta. Localization and quantitative expression of caprine PRPs were compared with bovine PRPs. The data indicate that *PRP *genes in caprine placenta have coordination functions for gestation, as they do in bovine. This is the first study of PRPs function in caprine placenta.

## Background

In ruminant placenta, various kinds of functional molecules such as steroid hormones, peptides, and prostanoids are expressed. These molecules contribute to a successful pregnancy and to the establishment of placenta. It is known that placental lactogen (PL) [1,2], pregnancy-associated glycoproteins (PAGs) [3-5], and interferon-τ (IFNT) [6-8] specifically appear in both caprine (goat) and bovine trophoblast. Prolactin-related protein (PRP) has been known as a placental-specific molecule that specifically expresses in trophoblastic binucleate cells in cattle. PRP is a gene of a non-classical member of the prolactin (PRL)/growth hormone (GH) family [9,10]. At least thirteen members are known to occur in cattle, and their nucleotide sequences are significantly analogous to PRL and PL in bovine species [11-13]. In rodents, diversity of the PRL family has been reported as well as the fact that they take on regulatory roles in pregnancy [14,15]. The placental PRL families in mouse and rat may play similar roles because of the evolutionary role of these genes in establishing pregnancy [15]. In the ruminants, bovines have some PRPs [12,13]; however there is no report on this in caprine species. We investigated the PRPs mRNA in caprine placenta. This paper introduces two novel types of cPRP genes that emerged from the cloning of a complete sequence of full-length cDNA and its expression. We named these sequences caprine prolactin-related protein-1 (cPRP1) and caprine prolactin-related protein-6 (cPRP6), based on their similarity to bovine PRPs.

## Results

### Sequences of *cPRP1* and *cPRP6* cDNA and deduced amino acids

**Figure 1:**
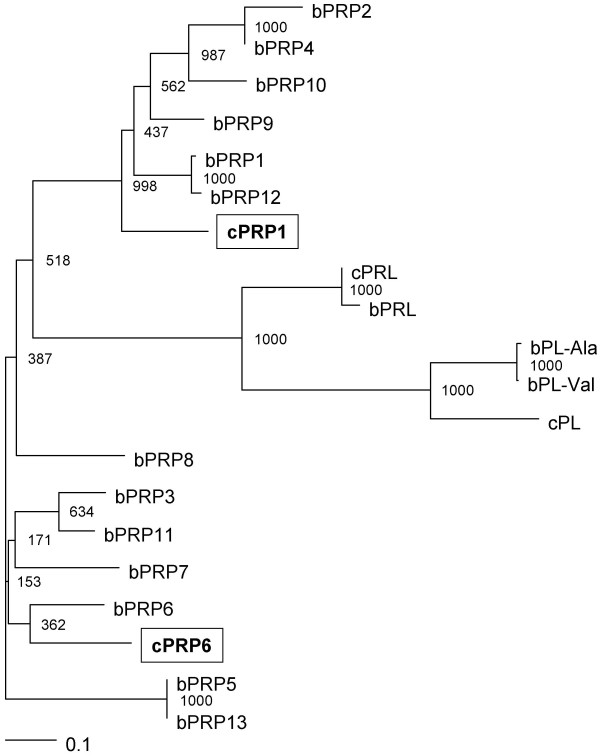
**Phylogenetic tree of prolactin and placental-prolactin family of caprine and bovine**. The tree was constructed using TreeView following the alignment of protein sequences given by the Clustal W 1.83 algorithm. The numbers at the base of each branch division represent bootstrap values after 1000 repeats. The scale bar represents 0.1 amino acid replacements per amino acid site. For GenBank/DDBJ accession numbers, refer to Materials and Methods.

**Figure 2:**
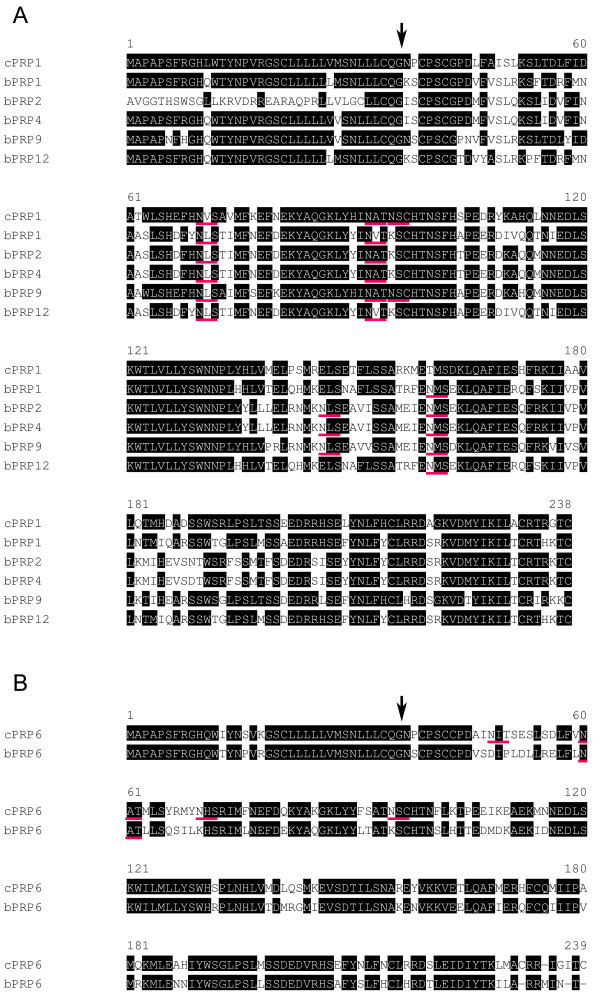
**Comparison of amino-acid sequences of (A) cPRP1 with bPRP1 cluster and (B) cPRP6 with bPRP6**. Residues identical to cPRP1 (A) or cPRP6 (B) are shown in black boxes. Amino acid sequences were aligned with assistance from Clustal W 1.83 on the DDBJ web site. The arrow indicates the putative primary cleavage site of the signal peptide of cPRP1 or cPRP6. The potential *N*-glycosylation site is underlined with a red line.

**Figure 3:**
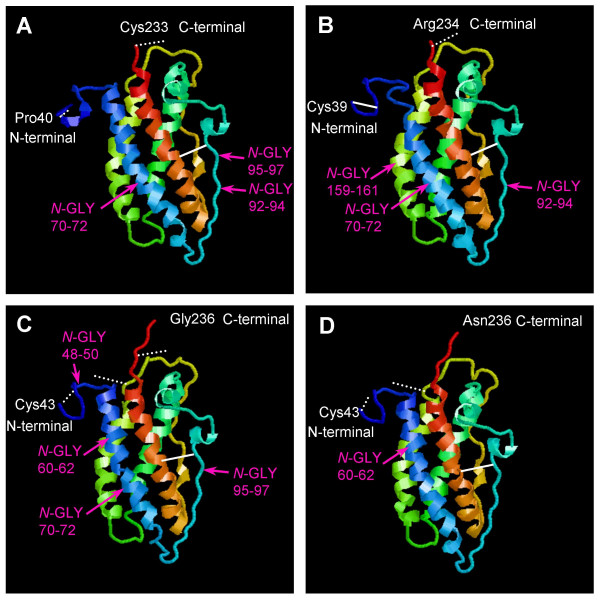
**Predicted 3D structure of (A) cPRP1, (B) bPRP1, (C) cPRP6 and (D) bPRP6 mature protein**. The 3D structures were predicted by the FAMS software. The cPRP1 structure was able to construct from Pro40 to the Cys233 amino acid region. The bPRP1 structure was able to construct from Pro39 to the Arg234 amino acid region. The cPRP6 structure was able to construct from Cys43 to the Gly236 amino acid region. The bPRP6 structure was able to construct from Cys43 to the Asn236 amino acid region. White solid lines indicate disulfide bonds. White dotted lines indicate predicted disulfide bonds. *N*-GLY refers to a potential *N*-glycosylation site.

Sequences of 933 and 957 nucleotides were isolated from caprine placentome and cloned as candidates of *cPRP1 *and *cPRP6*. The predicted protein sequence regions (CDSs) were composed of 717 nucleotides in *cPRP1 *and 720 nucleotides in *cPRP6*. The amino acid sequences deduced from full-length *cPRP1 *and *cPRP6 *cDNA are 238 and 239 amino acids (aa). The *cPRP1 *and *cPRP6 *sequences were submitted to the DNA Data Bank of Japan (DDBJ), and the DDBJ/GenBank/EMBL accession numbers are AB231295 and AB231296. A homology search can determine intermolecular similarity. It is also possible to learn the stage of the molecular evolution with the speed from the branching and distance in a phylogenetic analysis. We analyzed the evolutionary interrelationships using predicted amino acids (aa) sequences between the cPRPs and bPRPs (Fig. 1). The cPRP6 aa sequence was ~74% homologous to bPRP6 (Fig. 2B), so it was named cPRP6. In contrast, the sequence homology of cPRP1 is closer to a cluster of bPRP1, bPRP2, bPRP4, bPRP9 and bPRP12 (called the "bPRP1 cluster"). This aa sequence was ~72% homologous to bPRP1, 61% homologous to bPRP2, 72% homologous to bPRP4, 76% homologous to bPRP9, and 71% homologous to bPRP12 (Fig. 2A). The predicted cPRP1 sequence was closest to bPRP9, but it was located outside of the bPRP1 cluster according to the phylogenetic tree analysis. cPRP1 changed in the an earlier stage which was earlier than the bPRP1 cluster when, the amino acid evolution was decided from the phylogenetic analysis. Therefore, this novel caprine sequence was named cPRP1. The predicted 3D structures of cPRP1, bPRP1, cPRP6 and bPRP6 mature regions are illustrated in Fig. 3. The structural differences in the *N*-glycosylation site, the disulfide bond (-S-S-), and each atomic configuration were confirmed.

### Localized and quantitative mRNA expression

**Figure 4:**
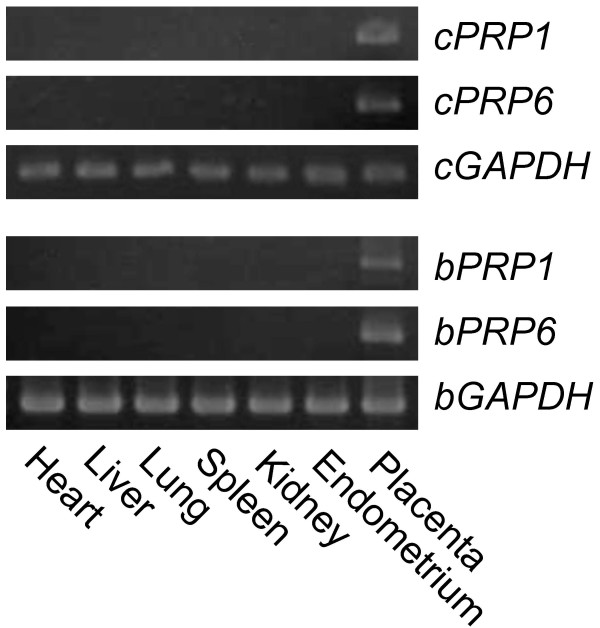
**Expression of *cPRP1*, *cPRP6*, *bPRP1 and bPRP6 *mRNA in caprine and bovine tissues**. Heart, liver, lung, spleen, kidney, and endometrium were used by RT-PCR. Caprine placentomal tissue at Day 50 of gestation or bovine cotyledonary tissue at Day 60 of gestation were used as placental samples. Caprine or bovine *GAPDH *expression in each tissue is presented as standard data.

RT-PCR analysis confirmed *cPRP1 *and *cPRP6 *expression in placenta such as *bPRP1 *and *bPRP6 *in bovine. No amplification was found in other caprine tissue, i.e. heart, liver, lung, kidney, spleen or endometrium (Fig. 4).

**Figure 5:**
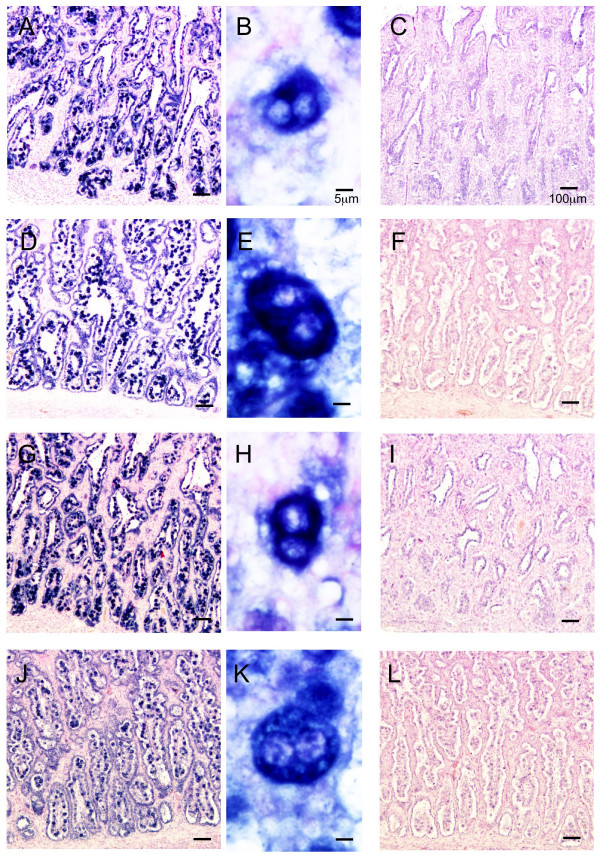
**Localization of *cPRP1*, *cPRP6*, *bPRP1 *and *bPRP6 *in the caprine and bovine placentome on Day 50 and 60 of gestation**. (A, B, C) *cPRP1*, (D, E, F) *cPRP6*, (G, H, I) *bPRP1*, and (J, K, L) *bPRP6 *mRNA were detected by *in situ *hybridization. (A, B, D, E, G, H, J, K) DIG-labeled anti-sense cRNA probes were used. (C, F, I, L) DIG-labeled sense cRNA probes were used. Seven micrometer sections of caprine and bovine placentome were hybridized with each probe. Scale bar = 100 μm on (A, C, D, F, G, I, J, L) and 5 μm on (B, E, H, K).

The mRNA localization was determined by *in situ *hybridization in the caprine or bovine placenta on day 50 (caprine) or 60 (bovine) of gestation (Fig. 5). Anti-sense RNA probes specifically detected mRNA transcripts in the placenta. All *PRPs *signals appeared in binucleate cells in the cotyledonary villous (Figs. 5A, B, D, E, G and 5H). No significant signals were detected with sense probes in any genes (Figs. 5C, F and 5I).

**Figure 6:**
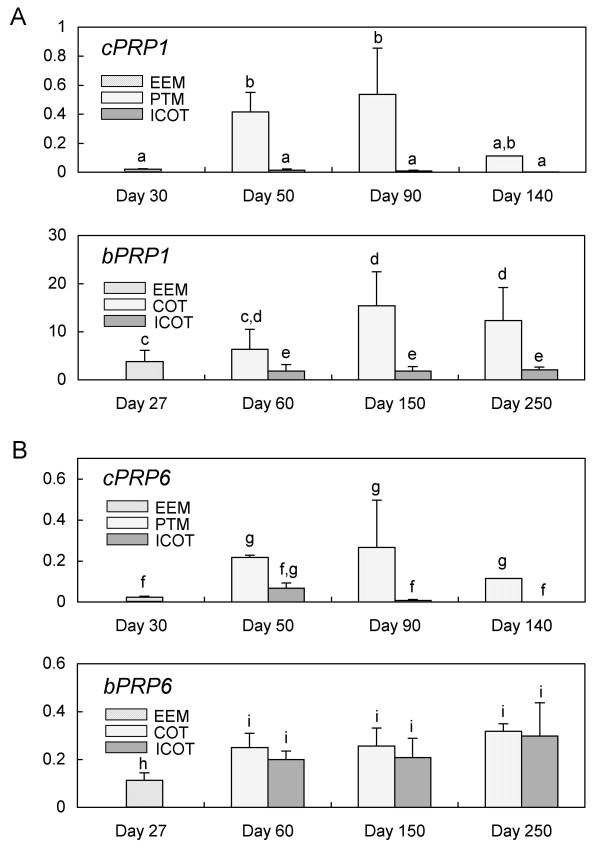
**Quantitative real-time RT-PCR analysis of (A) *cPRP1 *and *bPRP1*, (B) *cPRP6 *and *bPRP6 *mRNA in caprine or bovine placenta**. The total RNA of caprine was extracted from cotyledons containing extra-embryonic membrane (EEM), placentome (PTM), and intercotyledonary fetal membrane (ICOT) on Day 30, Day 50, Day 90 and Day 140 of gestation. The total RNA of bovine was extracted from cotyledons containing extra-embryonic membrane (EEM), cotyledonary placenta (COT), and intercotyledonary fetal membrane (ICOT) on Day 27, Day 60, Day 150, and Day 250 of gestation. The expression of these mRNAs was normalized to the expression of *GAPDH *measured in the corresponding RNA preparation. Values are mean ± SD. Values with different letters are significantly different (*P *< 0.05).

Quantitative real-time RT-PCR analysis showed that the *cPRP1 *and *cPRP6 *expression intensities increased from Day 30 to Day 50 and then remained constant to Day 90 and thereafter decreased until Day 140 in placentome (the cotyledonary and caruncular parts were not able to separate (PTM); see Fig. 6). In the caprine intercotyledon (the area between the cotyledonary villous (ICOT)), the *cPRP1 *expression intensity held steady from Day 30 to Day 90 and thereafter decreased until Day 140, although the intensity was low compared to that in PTM. The *cPRP6 *expression intensity increased from Day 30 to Day 50 and thereafter decreased until Day 140 in PTM, but rather low expressions were found in ICOT. The profile of *cPRP1 *was weaker than *bPRP1 *through the pregnancy. Especially *bPRP1 *intensively expressed in placentomal and interplacentomal tissues.

### Translation of *cPRP1* and *cPRP6*

**Figure 7:**
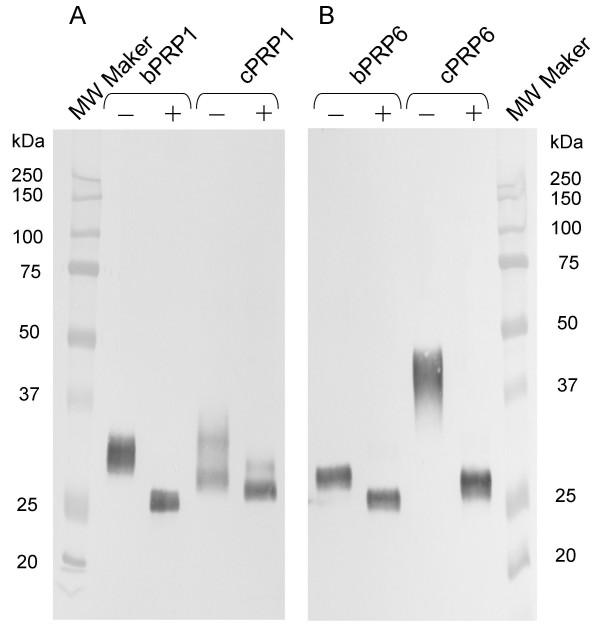
**Western blot analysis of recombinant FLAG-tag fusion (A) cPRP1 and bPRP1, (B) cPRP6 and bPRP6 proteins**. Conditioned media from HEK 293 cells transiently transfected with each gene were collected, and the purified proteins (1 ng) were loaded in separate lanes. The proteins were separated by SDS PAGE and specific proteins were detected by Western blot analysis using an anti-FLAG antibody. (-): Non *N*-degrycosylation treatment; (+): *N*-degrycosylation treatment; MW Marker: molecular weight marker.

Cloning sequences of *cPRP1 *and *cPRP6 *were effectively translated in an HEK293 cell system, like bovine *PRPs *(Fig. 7). Two different sizes of protein were translated of ~30 and 33 kDa with a FLAG-tag in cPRP1. Those translated-protein sizes were close to those in bPRP1 (Fig. 7A). However, translated cPRP6 was larger than bPRP6, at ~45 kDa and 29 kDa. There was a difference of >15 kDa between the bovine and caprine values. From the sequence analysis, there were no differences between them in predicted size (Fig. 7B). These PRPs were migrated to ~26 kDa by *N*-deglycosylation treatment (Fig. 7).

## Discussion

The diversity of the PRL gene family has been shown in mouse, rat and cattle; however, there exists only limited functional information except for PRL, PL and some rodents PLPs [11,14-18]. In particular, ruminants commonly have various genes of this family, but there is no information regarding goats, even though anatomical evidence shows a similarity to the placenta in ruminants. In cattle, thirteen varieties of PRP paralogs have been reported. In the present study, we identified novel PRPs in caprine and compared the properties of goat and cattle.

Novel *PRP1 *and *PRP6 *genes were detected and cloned from caprine placenta and their deduced amino acid sequences were determined to have some specific properties. Phylogenetic analysis suggests that cPRP1 separated from bovine PRP1 cluster in an early evolutionary stage (Fig. 1). In contrast, cPRP6 and bPRP6 molecules were phylogenetically adjacent (Fig. 1). This indicates that a primitive PRP6 molecule existed before it evolved into caprine and bovine.

The *N*-terminal regions of the bPRP1 and bPRP6 proteins were rich in hydrophobic amino acid residues, which is characteristic of the signal peptide [19,20]. The signal peptide sequence of cPRP1 and cPRP6, composed of 36 amino acids, is well conserved in bPRP1 and bPRP6. This information suggests that the signal cleavage site (between the Glu-36 and Arg-37) of cPRPs is the same as that of bPRPs (Fig. 2).

The bPRP1 mature protein is predicted to have three S-S bonds with six cysteines (Cys) at 39, 42, 97, 215, 232, and 238 (Figs. 2A, 3A, and 3B) [12,19]. The numbers and positions for these in the predicted cPRP1 exactly coincide with those of bPRP1. The 3D structure of cPRP6 was also similar to that of the mature bPRP6 protein (Fig. 3). bPRP6 is predicted to have three S-S bonds with six cysteines (Cys) in the sequence. However, cPRP6 mature protein is predicted to have eight Cys, with six Cys in aa positions at 39, 42, 43, 97, 174, and 215 corresponding with those in bPRP6 and an extra two in positions 232 and 239 (Figs. 2B, 3C, and 3D) [12,20]. However, these two last Cys were present in most other bPRPs, including bPRP1 (Figs. 2 and 3). Therefore PRP6 may have a highly homologous sequence in caprine and bovine, with a slight difference in 3D structure.

cPRP1 has two consensus sequences for potential *N*-glycosylation sites (Asn-X-Ser/Thr) at positions 70–72 and 92–94 (Figs. 2A and 3A), and an atypical *N*-glycosylation site, Asn-X-Cys was found at position 95–97. Typically, bPRP1 had three potential *N*-glycosylation sites at positions 70–72, 92–94 and 159–161 (Figs. 2A and 3B) [19]. So, the potential *N*-glycosylation sites are different in bovine and caprine, but the number of sites is the same, so they may translate a similar molecular size. This expectation was supported by the result of HEK 293 cell translation at 33 kDa (Fig. 7A).

cPRP6 had three consensus sequences for *N*-glycosylation at positions 48 to 50, 60 to 62, and 70 to 72 (Figs. 2B and 3C) with the atypical *N*-glycosylation site (Asn-X-Cys) at position 95–97 as in cPRP1. However, bovine PRP6 had only one consensus sequence for a typical potential *N*-glycosylation, at position 60–62. (Figs. 2B and 3D) [12,20]. This is a unique *N*-glycosylation position in caprine and bovine, which is not present in the cPRP1 and bPRP1 cluster genes (bPRP1, bPRP2, bPRP4, bPRP9 and bPRP12) (Fig. 1). *N*-glycosylation positions of 92–94 were confirmed in all PRP aa sequences except for caprine and bovine PRP6. In comparing the *N*-glycosylation sites in cPRP6 and bPRP6, the caprine may have four sites and bPRP, one site. Western blot analysis of an HEK293 cell system showed that cPRP6 migrated to 45 kDa and bPRP6 migrated to 29 kDa (Fig. 7B). Both *N*-degrycosylated proteins were migrated to ~26 kDa (Fig. 7B). The difference in the size of the proteins is related to the sugar-chain status.

Primary mRNA expression of *cPRP1 *and *cPRP6 *was observed in trophoblast binucleate cells (Fig. 5), and HEK293 cells translated these mRNA to each protein individually. Both *cPRPs' *mRNA were expressed in the extra-embryonic membrane after the implantation period (Fig. 6). In the bovine, *bPRP1 *and *bPRP6 *expression were found in the trophoblast binucleate cells (Fig. 5) and their expressions were kept from the implantation to late pregnancy in the placentomal villi (Fig. 6). The trophoblast binucleate cells are also primary expression cells for the *bPRPs *[21-25] and may have specific roles for the implantation and placentation of the fetomaternal interface [26-28]. The two caprine PRPs may also be related to implantation or placentation, like the bovine PRPs, since the localization and stage of expression of both caprine *PRP1 and PRP6 *were similar to those of bovine *PRPs*. The functional roles may not coincide between cPRP1 and bPRP1 (or cPRP6 and bPRP6), since the *PRP1 and PRP6 *expression profiles are different from those for bovine (Fig. 6). The bovine placental weight increases from the early to late stage of gestation [29]. In contrast, small ruminant placental weight increases to the middle stage of gestation (Days 70 to 80) and decreases to the late stage of gestation [30]. The change of the placental weight correlates with the placentation. The *bPRPs *expression appears to relate to the placentation in bovine ICOT. In contrast, the placentation decrease in the goat after the mid-pregnancy may be related to the low *cPRPs *expression in the ICOT.

It is important to confirm the caprine PRPs in order to understand the mechanism of implantation and placentation in small ruminant. Until now, the placental hormone in a small ruminant has been considered without PRP [31]. We suggest that PRP is also related to the placental-specific endocrine activity in a small ruminant.

## Conclusion

In conclusion, we have found two *PRPs *in caprine placenta. These *PRP *sequences had a high homology with the sequence of bovine *PRPs*. These *PRPs *were expressed in trophoblast binucleate cells as are bovine *PRPs*. They expressed from after the implantation period to late in gestation. These genes could independently produce mature recombinant proteins in a mammalian cell-expression system. We were able to specify the *PRP *genes, which was alike between the ruminants. Finally, we suggest that the caprine may have many more PRPs, recalling that thirteen PRPs or sixteen PLPs have been discovered in bovine or rodents.

## Methods

### Animals and tissues

Caprine placental tissues intended for cDNA cloning and mRNA expression were collected from Japanese Saanen goats. Extra-embryonic tissues and placenta were collected on days 26 to 30, 50 to 52, 86 to 92, and 135 to 139 after artificial insemination or natural mating (designated as day 1 of pregnancy). The tissues were obtained into two portions; the PTM and ICOT. It was difficult to divide the cotyledon (COT) and ICOT on days 26 to 30, and thus the COT contained very few villi. Tissues from three different goats on day 30 and one cow on day 26 of gestation (n = 4) were used as Day 30 extra-embryonic membrane (Day 30 EEM). Placentomal tissues were collected on days 50 (two samples), and 52 (two samples) (in total, n = 4) and designated as Day 50 PTM and ICOT. Sample materials from days 86 (one samples) and 92 (three sample) (in total, n = 4) and days 135 (one sample) and 139 (one samples) (in total, n = 2) were marked as Day 90 PTM, ICOT, Day 140 PTM, and ICOT. The collected tissues were stored at -80°C until RNA extraction. Some of the placentomes from day 50 were fixed in 3.7% formaldehyde PBS (pH 7.4), embedded in paraffin wax and stored at 4°C for *in situ *hybridization. Details of bovine placental-tissue collection have been provided in previous reports [21,22]. All procedures for these animal experiments were carried out in accordance with guidelines and ethical standards approved by the Animal Ethics Committee of the Laboratory Animals for Biomedical Research of the Graduate School of Agricultural Science, Tohoku University and National Institute of Agrobiological Sciences for the use of animals.

### Cloning of full-length *cPRP1* and *cPRP6* cDNA

The new full-length *cPRP1 *and *cPRP6 *cDNA was isolated from caprine cotyledonary tissue by the 3'-rapid amplification of cDNA ends (RACE) method. In brief, a complete RNA was isolated from a caprine placentome from day 50 of gestation using ISOGEN (Nippon Gene, Toyama, Japan). We performed 3'-RACE using a 3'-full RACE core set (Takara, Kyoto, Japan) with a *cPRP1*-specific forward primer (5'-CCACAGTCAACAGGAGTCCTC-3') and a *cPRP6*-specific forward primer (5'-CCAACAGAGAGTCCTCACCCTGCGA-3'). Both *cPRP *primers were designed from a bovine *PRP *sequence. The 3'-RACE products were sequenced using an ABI Prism 370 automatic sequencer (Applied Biosystems, Foster City, CA, USA) after cloning in a pGEM-T Easy Vector (Promega, Madison, WI, USA).

### Phylogenetic analysis

The deduced cPRP1 and cPRP6 protein sequences were aligned with bPRPs using the multiple-alignment software Clustal W 1.83 found on the DDBJ web site. Clustal W was also employed to calculate trees using the Neighbor-Joining (NJ) method [32]. TreeView was used to display the phylogenetic tree [33,34]. The values represent bootstrap scores for 1,000 trials, indicating the credibility of each branch. Except for the cPRP1 and cPRP6 sequences, the bPRPs and bPL protein sequences were obtained from GenBank. Their GenBank accession numbers are: bPRP1 (J02944), bPRP2 (M27239), bPRP3 (M27240), bPRP4 (M33269), bPRP5 (X15975), bPRP6 (AB245482), bPRP7 (AB187564), bPRP8 (AB196438), bPRP9 (AB204881), bPRP10 (AB255602), bPRP11 (BK005438), bPRP12 (BK005439), bPRP13 (BK005440), bPL-Ala (J02840), bPL-Val (M33268), bPRL (NM_173953) and cPRL (X76049). The cPL sequence was obtained from Sakal et al. [1].

### Three-dimensional structure prediction by FAMS

We predicted the three-dimensional (3D) structure of cPRP1, bPRP1, cPRP6 and bPRP6 using the FAMS (Fully Automated Homology Modeling System) [35,36]. FAMS is a software program that predicts 3D models for target proteins based on the structure of known proteins of high homology. For cPRP1, bPRP1, cPRP6 and bPRP6, the 3D structure was constructed based on the human prolactin (hPRL) 3D structure (Protein Data Bank ID: 1N9D). The FAMS program requires only an amino-acid sequence as input, and constructs 3D model structures automatically. Visualization of the 3D structure was performed using RasMol 2.7.3 software [37,38].

### RT-PCR

The tissue distribution of *cPRP1*, *cPRP6*, *bPRP1*, and *bPRP6 *expression was studied using RT-PCR. Caprine or bovine *GAPDH *was used as a positive control for the PCR. Details of the RT-PCR method have been described in previous reports [21,22]. The total RNA in a total reaction mixture was used for reverse transcription and template cDNA synthesis using oligo(dT) primer and Superscript III reverse transcriptase (Invitrogen, Carlsbad, CA, USA) at 50°C for 50 min. Each PCR contained a cDNA template, primers, deoxynucleotide triphosphate mixture (dNTP), MgCl_2_, 10 × PCR buffer II, autoclaved milliQ water, and AmpliTaq gold DNA polymerase (Applied Biosystems). Amplification conditions included denaturation at 95°C for 30 sec and extension at 72°C for 1 min. Twenty six cycles were performed for all samples. The annealing temperature was set at 60°C for 30 sec. A single denaturation step at 95°C for 10 min before the first PCR cycle and a final extension step at 72°C for 10 min after the last PCR cycle were also performed. The PCR products were analyzed by agarose-gel electrophoresis and visualized by ethidium bromide staining. The primers encoding for the *cPRP1*, *cPRP6, bPRP1*, and *bPRP6 *sequences were designed using our obtained sequences (caprine) and GenBank accession No. J02944 and AB245482 (bovine). The designated primers are listed in Table 1. All the primers were commercially synthesized (Tsukuba Oligo Service, Tsukuba, Japan).

**Table 1:** Oligonucleotide primers used for RT-PCR analysis

Gene	Primer	Sequence	Position
*cPRP1*	Forward	5' TGTCCCACGAGTTCCATAACGT 3'	237–258
(AB231295)	Reverse	5' GCACGCCAGGATCTTGATGTA 3'	742–722
*cPRP6*	Forward	5' TCACCTCAGAATCCCTTTCAGA 3'	38–59
(AB231296)	Reverse	5' CATGCCATGAGCTTGGTGTAA 3'	587–567
*cGAPDH*	Forward	5' GACCCCTTCATTGACCTTCAC 3'	1–21
(AJ431207)	Reverse	5' TCATAAGTCCCTCCACGATGC 3'	424–404
*bPRP1*	Forward	5' CTAATCTGCTCCTGTGCCAAGG 3'	173–194
(J02944)	Reverse	5' ATGACGCCTATCTTCAGCGCT 3'	705–685
*bPRP6*	Forward	5' TGTCCTGACGTGTCTGACATCC 3'	19–40
(AB245482)	Reverse	5' GCAGGCAGTGGAACAGGCTATA 3'	541–520
*bGAPDH*	Forward	5' CCTTCATTGACCTTCACTACATGGTCTA 3'	71–98
(U85042)	Reverse	5' GCTGTAGCCAAATTCATTGTCGTACCA 3'	927–901

### *In situ *hybridization

The full-length cDNA of *cPRP1*, *cPRP6*, *bPRP1*, and *bPRP6 *was used as a template for hybridization-probe synthesis. Complementary digoxigenin (DIG)-labeled antisense and sense RNA probes were prepared as described in previous studies [21,22]. The Day 50 caprine and Day 60 bovine placentomes were sectioned into 7 μm-thick sections for hybridization. *In situ *hybridization was performed using the automated Ventana HX System Discovery with a RiboMapKit and BlueMapKit (Ventana, Tucson, AZ, USA) [21,22]. Briefly, caprine and bovine sections were hybridized with DIG-labeled probes in a RiboHybe (Ventana) hybridization solution at 60°C (*cPRP1*), 63°C (*cPRP6*), 65°C (*bPRP1*), or 67°C (*bPRP6*), for 6 h. The sections were washed three times in RiboWash (Ventana) (at 60°C, 63°C, 65°C, or 67°C, for 6 min) after hybridization and were fixed in RiboFix (Ventana) (at 37°C, for 10 min). The hybridization signals were detected with monoclonal-anti-digoxin biotin conjugate (Sigma, Saint Louis, MI, USA) and the biotin localized with the alkaline phosphatase system. The hybridized glasses were observed after preparation with a Leica DMRE HC microscope (Leica microsystems, Wetzlar, Germany) with a Fujix digital camera HC2500 (Fujifilm, Tokyo, Japan).

### Real-time RT-PCR

Gene expression of *cPRP1*, *cPRP6*, *bPRP1*, and *bPRP6 *was confirmed quantitatively at each stage of gestation by real-time RT-PCR analysis using the SYBR Green assay. Fifty ng of the total RNA was reverse transcribed into cDNA for 30 min at 48°C by MultiScribe™ reverse transcriptase with a random primer, dNTP mixture, MgCl_2 _and RNase inhibitor. In the SYBR Green assay, primer pairs were designed using the Primer Express 1.0 software program (Applied Biosystems). The primers for each gene are listed in Table 2. Thermal-cycling conditions included initial-sample incubation at 50°C for 2 min and at 95°C for 10 min, followed by 40 cycles at 95°C for 15 sec and at 60°C for 1 min. The cycle-threshold values (C_T_) indicate the quantity of the target gene in each sample and were determined in real time using an ABI Prism 7700 sequence detector (Applied Biosystems). The relative difference in the initial amount of each mRNA species (or cDNA) was determined by comparing the C_T _values. The standard curves for each gene were generated by serial dilution of plasmid containing *cPRP1*, *cPRP6*, *cGAPDH*, *bPRP1*, *bPRP6*, or *bGAPDH *cDNA to quantify the mRNA concentrations. The ratios of *cPRP1*/*cGAPGH*, *cPRP6*/*cGAPGH*, *bPRP1*/*bGAPGH *and *bPRP6*/*bGAPGH *mRNA were calculated to adjust for any variations in the RT-PCR reaction. All values are presented as mean ± SD. Statistical analysis was performed using one-way ANOVA followed by the Tukey-Kramer multiple-comparison test. Differences were considered significant at *P *< 0.05.

**Table 2:** Oligonucleotide primers used for Real-time RT-PCR analysis

Gene	Primer	Sequence	Position
*cPRP1*	Forward	5' CAAATCTGCTCCTGTGCCAA 3'	132–151
(AB231295)	Reverse	5' AAGGAGATGGCGAACAAGTCA 3'	198–178
*bPRP1*	Forward	5' TCGTGTTGCTGTACTCCTGGAA 3'	458–479
(J02944)	Reverse	5' GGAAGGCGTTTGACAGTTCTTT 3'	541–520
*cPRP6*	Forward	5' GATATTTACACCAAGCTCATGGCA 3'	562–585
(AB231296)	Reverse	5' GGATGGCATGGATGTGGATT 3'	631–612
*bPRP6*	Forward	5' GGAGAATAATATTTACTGGTCGGGAC 3'	447–472
(AB245482)	Reverse	5' AAAATGCAGAATGACGCACATCT 3'	520–498
*cGAPDH*	Forward	5' GCCATCACCATCTTCCAGGA 3'	115–134
(AJ431207)	Reverse	5' CCACGTACTCAGCACCAGCA 3'	184–165
*bGAPDH*	Forward	5' AAGGCCATCACCATCTTCCA 3'	178–197
(U85042)	Reverse	5' CCACTACATACTCAGCACCAGCAT 3'	253–230

### Production and purification of recombinant proteins

The *cPRP1*, *cPRP6*, *bPRP1*, and *bPRP6 *sequences encoding the mature-protein region, which combined the FLAG and 6 × His epitope tag sequences, were inserted into the pFLAG-CMV-3 vector (Sigma). The constructed plasmid was transfected into HEK 293 cells using FuGENE 6 (Roche Diagnostics, Basel, Switzerland) for transient transfection. Stably transfected HEK 293 cells were adapted to the suspension culture in a spinner flask using 293 SFM II medium (Invitrogen, Gibco) and cultured in an atmosphere of 5% CO_2 _in air at 37°C for 3 days. The medium was separated by centrifugation.

Recombinant FLAG-tag and 6 × His-tag fusion proteins were purified using the 6 × His-tag portion. Approximately 1 liter of conditioned medium was processed at a time. A medium to which 1 ml Ni Sepharose 6 Fast Flow (Amersham Bioscience, Buckinghamshire, UK) was added was mixed and equilibrated with a 20 mM sodium-phosphate buffer, pH 8.0, containing 300 mM NaCl and 20 mM imidazole. Only the 6 × His-tag proteins bind to the Ni Sepharose 6 Fast Flow carrier. The medium with carrier was chromatographed on a PD-10 column (Amersham Bioscience). The fractions with carrier were washed in the 20 mM imidazole. The fractions were eluted by 250 mM imidazole. We also examined the cutting of the carbohydrate chain modified for the proteins using *N*-Glycosidase F Deglycosylation kit (Roche).

### Western blot analysis

One ng of purified proteins was loaded on each lane, separated by SDS-PAGE, and electrophoretically transferred onto a polyvinylidene-difluoride membrane [39]. Western blotting was performed using the method of Towbin et al. [40]. Briefly, the membrane was blocked in 10% skimmed milk overnight and incubated with mouse anti-FLAG M2 (Sigma) for 1 h at room temperature, followed by incubation with anti-mouse IgG conjugated with alkaline phosphatase (Sigma) (diluted 1:3000) for 1 h at room temperature. Immunopositive bands were stained using NBT (Bio-Rad, Hercules, CA, USA) and BCIP (Bio-Rad).

## Authors' contributions

KU participated in the design of the study, and carried out most of the experiments. TT participated and coordination in the design of the study, and performed the recombinant protein productions and Western blotting. KU, TT, MH, KK and KH collected the tissue samples of goats and cattle. YA, HS and ES carried out all animal care and tissues collection. KH participated and coordination in the design of the study, and helped to draft the manuscript. All authors read and approved the final manuscript.
